# Evaluation of Legume–Rhizobial Symbiotic Interactions Beyond Nitrogen Fixation That Help the Host Survival and Diversification in Hostile Environments

**DOI:** 10.3390/microorganisms11061454

**Published:** 2023-05-31

**Authors:** Ravinder K. Goyal, Jemaneh Z. Habtewold

**Affiliations:** 1 Agriculture and Agri-Food Canada, Lacombe Research and Development Center, Lacombe, AB T4L 1W1, Canada; 2 University of Lethbridge, Lethbridge, AB T1K 3M4, Canada

**Keywords:** abiotic and biotic stress, legume, rhizobia, stress tolerance, symbiosis, rhizosphere, microbiome, evolution

## Abstract

Plants often experience unfavorable conditions during their life cycle that impact their growth and sometimes their survival. A temporary phase of such stress, which can result from heavy metals, drought, salinity, or extremes of temperature or pH, can cause mild to enormous damage to the plant depending on its duration and intensity. Besides environmental stress, plants are the target of many microbial pathogens, causing diseases of varying severity. In plants that harbor mutualistic bacteria, stress can affect the symbiotic interaction and its outcome. To achieve the full potential of a symbiotic relationship between the host and rhizobia, it is important that the host plant maintains good growth characteristics and stay healthy under challenging environmental conditions. The host plant cannot provide good accommodation for the symbiont if it is infested with diseases and prone to other predators. Because the bacterium relies on metabolites for survival and multiplication, it is in its best interests to keep the host plant as stress-free as possible and to keep the supply stable. Although plants have developed many mitigation strategies to cope with stress, the symbiotic bacterium has developed the capability to augment the plant’s defense mechanisms against environmental stress. They also provide the host with protection against certain diseases. The protective features of rhizobial–host interaction along with nitrogen fixation appear to have played a significant role in legume diversification. When considering a legume–rhizobial symbiosis, extra benefits to the host are sometimes overlooked in favor of the symbionts’ nitrogen fixation efficiency. This review examines all of those additional considerations of a symbiotic interaction that enable the host to withstand a wide range of stresses, enabling plant survival under hostile regimes. In addition, the review focuses on the rhizosphere microbiome, which has emerged as a strong pillar of evolutionary reserve to equip the symbiotic interaction in the interests of both the rhizobia and host. The evaluation would draw the researchers’ attention to the symbiotic relationship as being advantageous to the host plant as a whole and the role it plays in the plant’s adaptation to unfavorable environmental conditions.

## 1. Introduction

In simplistic terms, a favorable environment of temperature, light, and nutrient availability is among the key factors that promote plant growth and their geographical distribution. Nonetheless, the territorialization of plants was an evolutionary process that started with the establishment of a set of plant species, followed by the diversification of another group of plants. Among the four phases of plant radiation [1], the last phase involved the diversification of angiosperms, which are the most dominant plant species and represent a vast ecological differentiation [2]. With over 350,000 species, angiosperms comprise about 90% of the total unique plant species [3,4]. What led to the success of angiosperms goes beyond the mutually beneficial animal–plant relationships to annual growth form, homeotic gene effects, asexual and sexual reproduction, a propensity for hybrid polyploidy, and an apparent high tolerance to extinction [3]. Many hypotheses have been formulated to explain the speciation and diversification of angiosperms. There is an uneven geographic distribution of plants, which could be due to the differential rate of diversification. It is believed that the key innovations, which are associated with plant morphological, physiological and behavior attributes, along with ecological opportunities, are the important determinants of a diversification rate [3,5].

With a count of 22,360 species [6], legumes are among the largest group of angiosperms, representing the outcome of a high diversification rate. Their adaptation to new climates and/or ecological niches, which are among the important factors promoting rapid diversification [7]), seems to have contributed to their occupancy in diverse habitats. By using the example of phaseoloid legumes that contain many commercial legumes, Li et al. [8] gathered more evidence favoring the interplay of ecological opportunities and key innovations in triggering diversification. While big climatic changes in the past shaped the diversification of plants, the current conditions of the environment determine their growth and distribution, especially of food and forage crops. Heavy metal toxicity, high and low temperatures, drought, salinity, extreme pH, pests, and pathogens all exert significant adverse impacts on the plant’s survival and productivity. Although plants have developed different mechanisms to cope with stress, those that fail become extinct in that environment.

Legumes develop a symbiotic interaction with rhizobia, which take the form of bacteroides and reside inside the nodular structures on the host roots. In exchange for carbon nutrients from the host, the rhizobial bacteria convert atmospheric nitrogen into its usable form, thus making the plant self-sufficient in nitrogen requirements [9]. Nitrogen is one of the macronutrients linked with plant growth and productivity. In non-leguminous crops, nitrogen is often supplemented in the form of synthetic fertilizers, which are now becoming an issue of environmental pollution and a threat to agricultural sustainability. The rhizobial symbiotic interactions evolved over time to provide the host with nitrogen and adaptability to varying environmental conditions and ecosystems [10,11]. It appears that nitrogen fixation is not the only driver of evolution, but host–symbiont genotype interactions and other factors do play an important role [12,13]. While rhizobial interactions have a direct positive impact on plants’ adaptability to both abiotic and biotic stresses, they can also have an indirect impact through the modification of the rhizosphere microbiome. The excretion of compounds (e.g., nodulation factors and exopolysaccharides) influences the structure of the rhizosphere, including non-symbiotic microbial communities, which in turn can alleviate stress and promote plant growth [14]. Hence, symbiotic relationships benefit legumes greatly, not only through nitrogen fixation but also through other benefits that aid the host’s survival and diversity in unfavorable settings. To deepen our understanding and inspire research for maximizing the benefits of symbiotic partnerships, this review attempts to give a detailed overview of these advantages. The review also focuses on how the presence of genetic mobile elements and the facilitation of their movement across distant bacterial species through horizontal gene transfer facilitate the acquisition of environmental and symbiotic adaptability.

## 2. Heavy Metal Stress Tolerance

Soil is a reservoir of several heavy metals that are required in microquantities to meet plants’ nutritional needs. Many of these metals, when present in higher concentrations due to either anthropogenic sources [15,16,17,18] or geological distribution, negatively affect plant growth and development. The metal stress leads to the formation of reactive oxygen species, which interfere with the structure and function of macromolecules, including lipids, proteins, and nucleic acids [19]. The metals in their high concentrations can also limit the rhizobial interaction with the host, resulting in a reduction of nodule number and nitrogen-fixating activity [20,21,22,23,24,25,26,27,28]. A constant threat to mutualism seems to have led to the development of metal-tolerating strategies in rhizobia. The bacteria isolated from heavy-metal soils often tend to tolerate relatively higher levels of the metals [29]. Numerous studies suggest that such rhizobia provide hosts with the ability to tolerate stress caused by many toxic metals, such as cadmium (Cd), nickel (Ni), mercury (Hg), chromium (Cr), arsenic (As), aluminum (Al), copper (Cu), lead (Pb), etc. (Table 1). There was a significant improvement in growth, development, and yield in several leguminous crops under heavy metal regimes when inoculated with appropriate rhizobial species. A symbiotic opportunity for legumes with naturally occurring metal-tolerating rhizobia allows them to take roots, thus promoting their diversification in such environments [30].

The genetic mechanism of the metal-tolerating ability of symbionts indicates an intricate network of multiple genes that relate to biosorption, localized accumulation, detoxification, RNA methylation, expression of antioxidant genes, hormone synthesis, and improving membrane stability. The presence of an efflux system, which reduces accumulation, has been a common strategy used by the rhizobia against several heavy metals. Some rhizobial symbionts have high Ni biosorption and storage capabilities, limiting their mobility in the plant [31,32]. A strong positive relationship between low concentrations of As in the shoots of *Medicago truncatula* and reduced expression of the plant’s NRT3.1-like gene, which is a nitrate transporter, has been reported [33]. The expression of this transporter gene was induced by abscisic acid, but ammonium, which is the fixed form of nitrogen in *Rhizobium*, had an antagonistic effect. The *Bradyrhizobium canariense* L-7AH strain that was isolated from a metal mining site effectively formed a symbiotic relationship with the legume *Lupinus albus* at high concentrations of Hg (up to 102 mg kg^−1^ vermiculite) with no apparent reduction in photosynthesis or nitrogenase activities [34]. The tolerance mechanism of the strain is not clear; however, in another study, the *Ensifer medicae* strain mediated an increase in mercuric reductase activity in *M. truncatula* nodules to convert the highly toxic mercuric cation to a less toxic volatile mercury metal [35]. A positive correlation between *Rhizobium*-induced differential methylation and expression of m6A RNA in soybean plants under Cd stress indicates a different mechanism of metal toxicity remediation [36]. Rhizobia-induced accumulation of Cu in *M. sativa* roots and increased expression of antioxidants have also been observed [37]. Another mechanism of excess Cu tolerance involves Cu homeostasis catalyzed by the multicopper oxidase CuO and a copper chaperone [38]. Legumes that can grow successfully in Al-stressed soil have evolved specific tolerance mechanisms such as prevention of metal uptake through the cell membrane and increased production of extracellular exopolysaccharides [39]. In a recent study, the differentially expressed genes under Al stress were linked to extracellular EPS, biofilm formation and cell membrane-stabilizing proteins in *Rhizobium phaseoli* [40].

With the recent advances in recombinant technology, genetic improvements in the symbiotic rhizobia for improved metal stress tolerance in legumes may be a way forward. In this context, the intrinsic abilities of rhizobial and non-rhizobial species that can tolerate metal-induced stresses may be considered as resources for the exploitation of novel metal-tolerant factors that can be used in different legumes and perhaps non-legume plants. For instance, genetically improved *Rhizobium pusense* KG2, a Cd^2+^ immobilizing strain, exhibited a substantial reduction in Cd absorption while enhancing root and shoot length, biomass, nitrogen contents, and superoxide dismutase activity [41]. After transferring the arsenite S-adenosylmethionine methyltransferase gene from *Chlamydomonas reinhardtii* into *Rhizobium leguminosarum*, there was an enhanced As tolerance in the *Rhizobium,* which methylated and volatilized the heavy metal [42]. This is an example that provides a sustainable remediation strategy for As-contaminated soils. Thus, recombinant DNA technology-based exploitation of metal resistance genes from other organisms, such as Cr resistance genes from *Akaligenes eutrophus* [43], is a promising technology for developing tolerant legumes of agricultural and ecological importance.

**Table 1:** A list of some symbiotic rhizobia that confers stress metal stress tolerance to legumes.

Symbiotic Rhizobia	Co-Inoculant	Legume Host	Metal	Beneficial Effects on the Plant	Reference
*Bradyrhizobium* sp. *RM8*		Greengram	Ni	Reduced uptake of Ni and Zn	[44]
*Rhizobium* sp. *RP5*		Pea		Reduced uptake of Ni and Zn	[45]
*Rhizobium TAL–1148*	*Bacilus subtilis*	Faba bean		Reduced uptake of Ni	[31]
*Rhizobium pisi*	*Ochrobacterium pseudogrignonense*	*Pongamia pinnata*		Ni accumulation in shoots and enhanced antioxidant activities	[46]
*Rhizobium pisi PZHK2*	*Ochrobacterium pseudo-grignonense PZHK4*	*Pongamia pinnata*		Enhanced activities of non-enzymatic antioxidants	[47]
*Bacilus japonicum CB1809*		Soybean	As	Enhanced production of growth-promoting hormones	[48]
*Rizobium* sp. *VMA301*		Black gram		As accumulation in roots	[24]
*Rhizobium. meliloti Rm5038*		*Medicago truncatula*		Lowered accumulation of AS in shoots	[33]
*Bacilus japonicum E109*	*Azospirillum brasilense Az39*	Soybean		Reduced As translocation to shoots	[49]
Recombinant *R. leguminosarum bv. trifolii*		Red clover		Alleviated As stress	[42]
*Sinorhizobium medicae*		*Medicago truncatula*	Hg	Alleviated Hg stress	[35]
*Bacilus canariense L-7AH*		White lupin		Limited mobility of Hg in roots	[34]
*Rhizobium leguminosarum RP 5*		Pea	Cd	Accumulated Cd in roots	[50]
*Sinorhizobium meliloti*		*Medicago sativa*		Enhanced absorption in roots	[51]
*Sinorhizobium fredii HH103*		Soybean		Modulated methylation and expression of m6A RNA	[36]
*Rhizobium pusense KG2*		Soybean		Reduced Cd^2+^ absorption	[41]
*Mesorhizobium strain RC3*		Chickpea	Cr	Reduced Cr uptake	[52]
*Rhizobium* sp. *AS05*	*Bacillus* sp. *AS03*	Horse gram		Reduced shoot translocation	[53]
*Rhizobium loti*		*Lotus purshianus*	Cu	Accumulation of Cu	[54]
*Sinorhizobium meliloti*		*Medicago sativa*		Increased antioxidant activities	[55]
*Sinorhizobium meliloti*		*Medicago sativa*		Reduced Cu translocation	[37]
*Rhizobium* spp.		*Phaseolus vulgaris*	Al	Production of exopolysaccharides	[39]
*Bacilus* sp. *750*	*Pseudomonas* sp., *Ochrobactrum cytisi*	*Lupinus luteus*	Pb	Reduced metal accumulation in shoots and roots	[56]

## 3. Tolerance to Drought, Salinity, and pH

Stresses caused by environmental factors such as drought, salinity, heat, and extremes of pH are among the major factors affecting plant growth and development. These environmental factors may exacerbate with the changing climate, thereby causing an adverse impact on agricultural production. The rhizobial symbionts that can confer legumes with tolerance against different types of stresses have been summarized in Table 2.

Drought reduces transpiration and water movement in legumes, thus restricting the circulation of nitrogen fixation products and inhibiting nitrogenase activity [57,58,59,60]. It also reduces biomass and chlorophyll contents and accumulates reactive oxygen species that can disrupt the functioning of different biomolecules including DNA [61,62,63]. Production of antioxidants (e.g., superoxide dismutase, catalase, ascorbic acid, and glutathione) and osmoprotectants (molecules that maintain the balance of osmotic potential in cells) are among the common responses to drought stress in most legumes [62]. There are many examples of rhizobial symbionts that confer drought tolerance to legumes, such as *B. diazoefficiens* SEMIA 5080 in soybean R01-581F, *Mesorhizobium huakuii* 7653R in *Astragalus sinicus* L., and *S. medicae* or *S. meliloti* in *M. truncatula* [64,65,66]. Inoculation of *R. meliloti* in kidney bean, black bean, mung bean, and chickpea increased the number of nodules and improved photosynthesis under water-deficient conditions [67]. Similarly, *S. fredii* strain SMH12 was shown to improve the number of nodules and the water potentials in soybean grown under drought stress [68]. Rhizobium-induced increases in antioxidant enzyme production [69], accumulation of osmoprotectants including proline and soluble sugars in nodules and roots [70], or genes that encode enzymes involved in trehalose synthesis [71] were associated with drought stress.

Soil salinity, which is strongly related to drought and gets intensified with the use of saline water for irrigation [72,73,74,75], is among the key factors affecting the efficiency of legume–rhizobia symbiosis [76]. It causes the accumulation of toxic ions in soil [77] and is correlated with poor-quality flavonoids in the root exudates of legumes, which affect the production of nod factors [78]. By influencing the early stage of legume–rhizobia interaction involving chemical communication and colonization or infection of root hairs, salt stress can result in poor establishment of legume–rhizobia symbiosis. The reduction in rhizobial infections under salt stress was observed in many legumes such as bean [79], soybean [80], pea [81], and chickpea [82], which resulted in reduced nitrogen fixation [83]. Some rhizobia are able to modulate the host’s response to salinity by inducing indole-3-acetic acid production and accumulation of osmoprotectant molecules [84], increasing root osmotic water flow via reducing xylem osmotic potential and increasing the amount of aquaporins [85], and changing the protein profile of the host plant [86]. It is unclear if the production of nod factors under high salt conditions would be as effective as in the normal situation although some similarities have been noticed [87].

pH is known to influence soil properties and nutrient availability, and hence the functioning of the soil microbial community. Most soil microbes including root-nodulating rhizobia prefer a near-neutral pH, whereas a large proportion of the global arable land is either acidic or alkaline [88]. Extreme pH conditions can affect the establishment of legume–rhizobia symbiosis [89], as a delay in nodulation under acidic conditions has been observed in many legume plants [90]. The fact that supplementation of molecules such as genistein, a nod gene inducer that reverses the effects of acidic conditions on the establishment of legume–rhizobia symbiosis [91], suggests that expression of symbiosis signals is influenced by pH [92,93,94]. Soil pH can also influence the structure of the rhizosphere community [95], which can have a significant influence on plant roots [96,97]. The legumes thriving in acidic soils have evolved tolerance mechanisms for soil acidic conditions through the production of nod factors that are different from those produced under neutral pH conditions [98]. The studies indicate a role for the rhizobia-specific genes actA, typA, atvA, lpiA, and ubiF in improving acid stress tolerance and symbiotic competitiveness [99,100,101,102]. *R. tropici* CIAT899, a highly acid-tolerant strain [103], induces the production of glutathione [104]. The bacterium could produce more (~1.8-fold) Nod factors in acidic than neutral growth conditions, and about half of them were different from the normal profile [98]. The rhizobial strains have displayed tolerance to conditions ranging from highly acidic [105,106] to highly alkaline [107]. The defense response to high pH includes an increase in antioxidants, organic acid production, and changes in certain proteins [108].

Species that are naturally tolerant to environmental stress could be exploited for developing tolerant rhizobial strains. Alternatively, a genetic engineering route could be adopted for strain improvement, as has been demonstrated through overproduction of cytokinin, trehalose-6-phosphate synthase, 1-aminocyclopropane-1-carboxylic acid deaminase, high-affinity cytochrome cbb3-type oxidase, indole-3 acetic acid, and flavodoxin [109,110,111,112,113,114].

**Table 2:** Symbiotic rhizobia that confer environmental stress tolerance to legumes.

Symbiotic Rhizobia	Co-Inoculants	Legume Host	Stress	Beneficial Effects on the Plant	Reference
*Mesorhizobium huakuii strain 7653R*		*Astragalus sinicus*	Drought	Improved N fixation and NH_4_^+^ assimilation	[65]
*Sinorhizobium medicae or S. meliloti*		*Medicago truncatula*		Enhanced allocation of reserves to osmolytes	[66]
*Sinorhizobium meliloti*		Kidney bean, black bean, mung bean, and chickpea		Improved nodule number and photosynthesis	[67]
*Rhizobium meliloti*		*Medicago sativa*		Enhanced antioxidants	[69]
*Sinorhizobium fredii strain SMH12*		Soybean		Improved nodule number and water potentials	[68]
*Rhizobium leguminosarum*		Faba bean		Enhanced production of osmoprotectants	[70]
*Rhizobium tropici CIAT 899*	*Paenibacillus polymyxa* spp.	*Phaselus vulgaris*		Increased leaf abscisic acid content	[115]
IAA-overproducing *Ensifer meliloti 1021 (Ms-RD64)*		*Medicago sativa*		Enhanced production of low-molecular-weight osmolytes	[109]
*Bradyrhizobium* sp. *SUTN9-2*		Mung bean		Enhanced ACC deaminase activity	[110]
*Rhizobium etli*		*Phaseolus vulgaris*		Overexpressed Trehalose-6-Phosphate Synthase	[111]
*Rhizobium etli*		*Phaseolus vulgaris*		Enhanced expression of Cytochrome cbb(3) oxidases	[112]
*Sinorhizobium meliloti*		Alfalfa		Overexpressed cytokinin and antioxidant enzymes	[113]
*Bradyrhizobium RJS9-2*		*Stylosanthes guianensis*	Salinity	Induced IAA production, enhanced osmoprotectant accumulation	[84]
*Rhizobium leguminosarum*		*Phaseolus vulgaris*		Contributed to enhanced root osmotic water flow	[85]
*Rhizobium phaseoli M1, M6, and M9*	*Pseudomonas* spp.	Mung bean		Expressed ACC deaminase	[116]
*Mesorhizobium ciceri IC53*	*Bacilus subtilis* NUU4	*Cicer arietinum.*		Increased proline contents	[117]
*Rhizobium meliloti*		*Medicago sativa*		Modulated key plant processes (efficient use of resources, oxidative stress, ion homeostasis)	[87]
*Sinorhizobium meliloti*				Overexpressed flavodoxin (Cyanobacterial origin)	[114]
*Rhizobium tropici CIAT899*		*Phaseolus vulgaris*	pH	Modulated rhizobial nod factors production	[98]
*Rhizobium tropici CIAT899*		*Phaseolus vulgaris*		Induced production of glutathione in beans	[118]
*Sinorhizobium meliloti*		*Medicago sativa*		Adaptive acid-tolerance response	[105]
*Rhizobium* spp.		*Medicago sativa Longmu 806*		Antioxidants and organic acids production	[108]

## 4. Protection against Diseases

Plants actively recruit a collection of microbes from the soil that expand the plant’s genomic and metabolic capabilities. The rhizosphere so formed can act as a microbial-mediated suppressor of soil pathogens. The suppression could be due to the plant-associated microbiome’s ability to deter the establishment of a pathogen through competition for nutrients and space, or it could be mediated through an antagonistic effect on a pathogen [119]. The rhizosphere provides a first line of defense for the plant. The pathogens that can penetrate this outer defense then encounter the plant’s innate defense, which could be basal or inducible. In the basal defense, small peptides that possess antimicrobial activities play an important role [120], and the inducible defense, which is very different from the basal defense, is a type of hypersensitive response involving perception of a pathogen, signal relay, strengthening of cell wall structures, and synthesis of antagonistic compounds [121]. The plant response against pathogens is augmented by endophytes. This has been demonstrated in an investigation where the microbial community enriched with families belonging to Chitinophagaceae and Flavobacteriaceae residing inside the host root was shown to possess suppressive activity against fungal root diseases [122]. The research provided insight on how endophytes can mount a defense against fungal pathogens. Flavobacterium harbors gene clusters encoding the production of non-ribosomal peptide synthetases and polyketide synthases that play an essential role in protection. The evidence was collected on a non-legume–endophyte interaction; however, it lends support to the presence of disease suppressive activity in other host–endophyte interactions. A 99% reduction in white rot incidence caused by a necrotrophic fungus, *Sclerotinia sclerotiorum*, was observed in Brassica when *M. loti* was co-inoculated with other PGPR species [123]. Another soil borne fungus, *Sclerotium rolfsii*, was inhibited from causing stem rot disease by the co-inoculation of groundnut with *Rhizobium* and *Trichoderma harzianum* [124]. It is unknown, though, if *Rhizobium* alone would have prevented the disease. Nevertheless, the inhibitory activity of certain *Rhizobium* strains was observed in cell-free cultures. The latter reduced the radial growth of *Macrophomina phaseolina*, *Rhizoctonia solani*, *Fusarium solani* and *Fusarium oxysporum* under in vitro conditions and suppressed root rot in soybean [125]. Similarly, *R. leguminosarum bv. phaseoli* isolates were able to inhibit the mycelial growth of soil-borne fungi causing root rot [126]. In another study, the biocontrol potential of *Rhizobium* and *Bradyrbizobium* against soil-borne root rot-causing fungi was observed in both leguminous (soybean and mung bean) and non-leguminous (sunflower and okra) plants under in vitro and field conditions [127]. More examples of symbiotic biocontrol potential against soil-borne fungal pathogens have been comprehensively reviewed [128]. The protection provided by a symbiotic interaction appears not to be a universal phenomenon. Our results on *R. leguminosarum* strain inoculation did not show inhibition of the root rot disease caused by *Aphanomyces euteiches* and *Fusarium avenaceum* in pea under controlled environmental conditions (unpublished). Although the inoculated plants were apparently healthier than the uninoculated controls, the minor effect was due to the robust growth of the nodulated plants rather than the inhibitory activity of the symbiotic bacterium. Host–bacterial interactions are complex; their protective antagonistic effect on pathogens may depend on a unique relationship determined by the host genotypes and bacterial strains [129].

Protection by a symbiotic Rhizobium–legume association has also been observed against bacterial and viral pathogens. Common bean plants inoculated with *Rhizobium etli* demonstrated strong resistance to *Pseudomonas syringae pv. phaseolicola*, as evidenced by a reduction in lesion size and pathogen count [130]. In this case, the resistance was linked to the accumulation of reactive oxygen species and enhanced callose deposition, which are typical characteristics of a hypersensitive response. It was speculated that *Rhizobium* inoculation primed the host plant against the pathogen. The activation of plant defense by the symbiotic bacteria co-inoculated with PGPR was earlier reported in pigeon pea [131]. There was an increase in phenyl ammonia lyase, peroxidase and phenol oxidase activities with a simultaneous decrease in pathogen β-1,3-glucanase and polymethyl galacturonase levels. The first three enzymes catalyze the synthesis of phenolic compounds with an antagonistic effect, and the last two enzymes aid the pathogen in degrading the plant’s structural components. The infection resulted in a systemic response in the plant with elevated levels of phenols in the leaf. The disease-tolerating effect of Rhizobium–host interaction was also observed against another bacterial pathogen, *Xanthomonas axonopodis*, which is responsible for common bean blight [132]. The protection was conferred both in greenhouse and field conditions. A strain of *R. leguminosarum bv. viceae* was able to protect faba beans against bean yellow mosaic potyvirus [133]. There was an increase in salicylic acid and peroxidase activity in leaves, thus suggesting the induction of a systemic resistance. According to a report, *Agrobacterium radiobacter* could reduce an early root infection by the potato cyst nematode *Globodera pallida* [134].

The nodulating hosts synthesize nodule-specific cysteine rich (NCR) peptides, which belong to a superfamily of defensins. The defensins contain conserved cysteine disulfide bridges that stabilize their structure, which is an important component of the structure–activity relationship [120,135]. The defensins display a wide-spectrum toxicity against bacterial, fungal, and viral microorganisms [120,136]. Some of the NCR-peptides are involved in bacteroid differentiation and survival [137,138]. In *M. truncatula*, more than 700 NCR-peptide genes are present. The obvious question of why the genome supports so many NCR-peptide genes and what role they play remains unanswered. Because defensins are known to exert a toxic effect against a diverse range of microbial pathogens, there is a possibility that these peptides may help to keep away the pathogens during the infection thread or symbiotic interaction. There is no direct evidence available yet for this viewpoint.

**Figure 1:**
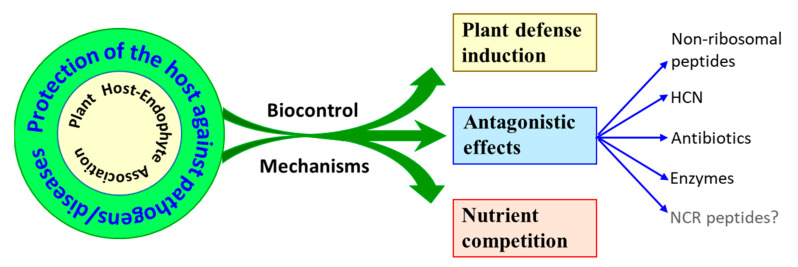
Illustration depicting rhizobia-related activities that confer legumes with tolerance against phytopathogens.

The rhizobial symbiotic interaction can assist the host in combating biotic stress in many ways (Figure 1). In addition to inducing the host plant’s defense, it can produce compounds antagonistic to pathogen growth and survival. The production of HCN, antibiotics, or enzymes that can degrade the fungal cell wall has been reviewed elsewhere [128]. An indirect biocontrol effect of rhizobia can also be mediated by creating an unfavorable nutrient environment for the pathogens. Many rhizobia produce siderophores that help sequester iron from the soil, which enables the host plant to survive and grow but exacerbates the nutrient deficiency, thereby negatively affecting the colonization of pathogens.

## 5. Role of Soil and Rhizosphere Microbiomes

Root nodulating rhizobia are not alone in the rhizosphere, which is a hotspot for biochemical activities derived by organisms from different kingdoms. Thus, it is highly likely that the metabolic efficiency of one organism depends on the other occupants of the rhizosphere. Plant–microbe interactions in the rhizosphere are very complex, and the mechanistic understanding of how different microbes influence each other’s functions is very limited. The technical advances of the recent past have provided a valuable tool in the form of high-throughput sequencing, which can take a snapshot of the microbiome composition. The accumulating information would help delineate the structure and functional relationships of a diverse microbial population. It is becoming evident that such relationships play a role in the alleviation of stress, thereby providing a more conducive environment for plant establishment and diversification.

Although rhizobial symbionts dwell inside the root nodules of legumes, their functional efficiency is not completely independent from the rhizosphere and soil microbiomes [95,139]. The structure of rhizosphere microbial communities in terms of both quality and quantity is not only influenced by the root exudates of the host plant but also by the microbial mineralization of soil nutrients [140]. Under normal rhizosphere conditions, a shift in the community composition of microbes can be associated with changes in the relative abundance of a few taxa (<5%), often called keystone taxa, which may have dominant and strong connections within the rhizosphere communities [141]. The members of Rhizobiales, which comprise *Rhizobium* and *Bradyrhizobium* spp., are among those that have been proposed as keystone taxa in different ecosystems [142]. This is supported by the enhanced abundance of beneficial bacteria and improved co-occurrence networks in the rhizosphere, and the shift in the structure of rhizosphere fungal communities after inoculation with symbiotic rhizobia [13,143]. Multiple factors may contribute to the change in rhizosphere population. The chemical signals released to attract the symbiotic species could be one of the determinants. It could also be due to a direct inhibitory effect as trifolitoxin produced by the *R. leguminosarum trifolii* CE3 strain was found to be associated with a reduction in the diversity of Proteobacteria [144]. An increase in ATP-binding cassette transporters in the rhizosphere when the black locust plant was inoculated with the phytoremediating *M. loti* HZ76 strain suggests an interplay of interactions that favors a buildup of stress-alleviating microbes [145]. There was a noticeable change in the rhizosphere microbiome when soybean plants were inoculated with a *B. diazoefficiens* USDA 110 strain defective in noel gene, which encodes an enzyme for fucose methylation of Nod factors [146]. In the study, a significant reduction in flavonoid exudation and root nodulation led to decreased bacterial diversity in the rhizosphere, co-occurrence networks, and depletion of root microbes. Further studies are needed to understand the cause of the change, whether the flavonoid exudation acts as a signal or nutrient, or if there is yet another mechanism. The role of host plants in determining the rhizosphere microbiome goes beyond the metabolic profile of root exudates. The plants establishing symbiosis with rhizobia also harbor other microbial species; the extent and type of such interactions could affect the microbe structure. *M. truncatula* mutants that were unable to establish arbuscular mycorrhizal (AM) symbiosis altered the microbial abundance in the rhizosphere [147]. Moreover, the AM-conditioned microbial community was able to promote nodulation in different legume plants in native soil. The shift in a microbial population, or lack thereof, can have a profound impact on the symbiotic relationship and plant growth, especially under stressful environmental conditions.

There have been numerous examples demonstrating the beneficial effect of non-rhizobial microbes improving the symbiotic performance of nodulating rhizobia (Table 1 and Table 2). Such collaborations have been successfully used to improve the stress tolerance capacity of rhizobia and leguminous plants. A substantial increase in Ni tolerance (600 mg Ni kg^−1^) was observed in faba bean plants when the *Rhizobium* TAL–1148 strain was co-inoculated with Ni-tolerant *Bacillus subtilis* [31]. A similar effect of metal toxicity mitigation was noticed in the *Pongamia pinnata* plant with the combined interaction of certain *R. pisi* and *Ochrobacterium pseudogrignonense* strains [46,47]. A high level of Ni in plant shoots was accompanied by an increase in the activities of catalase, superoxide dismutase, peroxidase, and ascorbate and an accumulation of antioxidant metabolites such as glutathione, proanthocyanidin, ascorbic acid, and flavonoids. These antioxidants and enzymes participate in the metabolism of reactive oxygen species that are generated as a plant response to various stresses. The collaborative advantage of symbiotic and non-symbiotic bacteria has been shown to ameliorate the survival and growth of other legumes under higher levels of several heavy metals, including As, Cr, Cd, Cu, Al, Pb, etc. Co-inoculation of *B. japonicum* E109 and *Azospirillum brasilense* Az39 to *Glycine max* cv. DM 4670 exhibited improved tolerance to As [49], and horse gram plants performed better against a high concentration of Cr in the presence of *Rhizobium* sp. AS05 and *Bacillus* sp. AS03 [53,148]. Contrary to the potential mechanism at work to endure the Ni stress, the toxic effect of the metals was minimized in these investigations through their accumulation in roots and limiting their translocation to aerial plant parts. Before building up in the plants, the extremely toxic Cr^6+^ may have been converted into the less toxic Cr^3+^ form [149]. The partnering bacteria could also reduce the bioavailability of the metal as there was a decreased accumulation of Pb and other metal contaminants in the shoots and roots of Yellow lupines with co-inoculation of *Bradyrhizobium* sp. 750 and plant growth-promoting rhizobacteria [56]. Microbial co-inoculants that can contribute to environmental stress tolerance in legumes have also been reported. For instance, co-inoculation of *Rhizobium* and two *Paenibacillus polymyxa* strains into drought-stressed bean plants exhibited improvements in the growth, N content, phytohormones, and nodulation of the plant [115]. Rhizobia-induced responses to salinity stress (e.g., inducing indole-3-acetic acid production, accumulation of osmoprotectant molecules, and increasing root osmotic water flow) were found to be contributed by non-rhizobial plant growth-promoting rhizobacteria and endomycorrhiza [150,151,152], suggesting the presence of yet unidentified non-rhizobial species that can be used as co-inoculants for improving salt tolerance in legumes.

The studies suggest that the host plant and the symbiotic *Rhizobium* species alone are not enough for a perfect symbiosis. The rhizosphere and soil microbiomes that contribute to defining the terrestrial ecosystem play an important role in plant growth and diversification. The symbiotic rhizobia not only supply nitrogen and confer stress tolerance to legumes, but they also maintain the microbial ecology of the rhizosphere, thereby increasing the plant’s fitness and adaptability to a wide range of environmental conditions. This interdependence, which shapes the microbial community, is an important determinant of the niche of native strains. A significant impact of the non-nitrogen-fixing microbial community on the legume–rhizobia symbiosis partly explains the often poor performance of the genetically modified strains over the native ones [151,152,153]. In addition, it has been noticed that legume plants that are grown in a rhizobia-free environment do not perform well even if they are supplemented with nitrogen fertilizer. By gaining in-depth knowledge of the complex interactions in a microbiome, the elite strains could be effectively utilized in commercial applications.

## 6. Evolution of Rhizobia for Increased Environmental and Symbiotic Fitness

Endosymbiosis is an example of evolution that directly led to the emergence of new physiological interactions, tissues, organs, and even new species (Ref. in [154]). The authors have provided a comprehensive review of how free-living bacteria, legumes, and rhizobia co-evolved to develop a partnership for nitrogen-fixing capability. It is estimated that legume–rhizobia symbiosis evolved about 55–60 Mya ago [155]. Nitrogen fixation and infection thread genes are often present on mobile genetic elements, which can be transmitted vertically with cell division or through horizontal gene transfer (HGT). The mobile elements, which could be insertion sequences, plasmids, transposons, pathogenicity islands, etc., play an important role in bacterial evolution and environmental fitness. Further, the recombination events, gene paralogy, and jumping of mobile elements within the genome supported the genomic diversity in rhizobia [156]. Although HGT played a dominant role in the transfer of key symbiosis genes and rhizobial genetic diversity, genome innovation and the reconstruction of regulatory networks were necessary for the functionalization of transferred genes [11]. There has been a continuous evolution of symbiosis genes that has resulted in rhizobial diversity ranging from high host specificity to promiscuity [157,158,159]. Genomic islands that improve bacterial fitness could be referred to as fitness islands, which may provide environmental fitness or symbiotic/pathogenic fitness during the bacterium’s interaction with the living host [160]. After integration and regulatory rebuilding, these fitness islands carrying novel adaptive genes can improve the fitness of recipient rhizosphere bacteria, including rhizobial symbionts. The discovery of many metal resistance genes (Cd, Ni, Zn, and Co) on mobile genetic elements in *Pseudomonas putida* KT2440 strain [161] illustrates the mechanistic potential for rhizobia to develop tolerance to heavy metals in contaminated soils. Similarly, stress-tolerant genes that can confer tolerance to high temperature and pressure have been found on the genomic islands in beta- and gamma-Proteobacteria [162]. Recently, a study proposed that the phenomenon of HGT is not only restricted to bacterial species but that gene transfer can also take place between plants and their microbiota [163]. In the study, which is yet to be peer reviewed, a trail of gene transfer events has been detected in *Arabidopsis thaliana* and its microbiome. The observation gains more support from the genome mining data, which suggest that abiotic stress resistance genes in plant genomes were acquired from microbes via HGT [164]. The detection of mobile elements carrying adaptive genes in the rhizosphere microbiome could be very challenging. However, the advent of high-throughput genome sequencing and bioinformatics tools has simplified the task of identifying probable insertion sites [165]. A hypothetical probability nevertheless requires rigorous biological validity. The studies suggest an important role of HGT in strain adaptation, which might have occurred over a long period of time. In the context of legume–rhizobial interactions, the improvement of strains based on HGT will not be without significant challenges. A thorough understanding of the favorable recipient and donor and the mechanism of HGT would be required, which would open new opportunities for strain and crop improvement.

## 7. Conclusions and Future Perspectives

**Figure 2:**
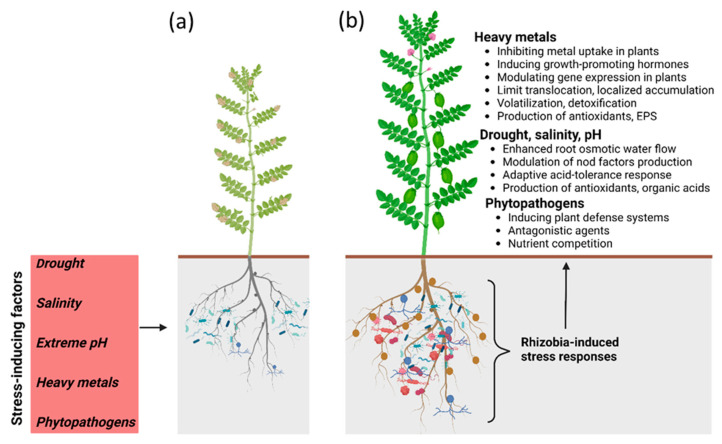
An illustration depicting factors that cause stressful conditions for the growth and development of legumes (**a**), and rhizobia-induced responses that help confer tolerance against stress in legumes (**b**).

Nitrogen fixation by symbionts in exchange for energy-rich carbon sources might have been the principal factor in the evolution of a symbiotic interaction involving plants and bacteria. Symbiotic fitness, however, is not only determined by the efficiency of nitrogen fixation but also by the combined defense of the host and the symbiotic bacterium against a wide range of environmental and biotic threats. The symbionts have evolved to tolerate adverse conditions, both natural and arising from anthropogenic activities. Numerous examples of legume–rhizobial interactions increasing the plant’s tolerance to a diversity of stresses (summarized in Figure 2) support this observation. The added advantages of a stronger defense and self-sufficiency in nitrogen have been important in legume expansion and diversification.

Significant progress, on how the symbiotic- or non-symbiotic bacteria acquired the stress tolerating ability, has been made. The rhizosphere microbiome was identified as a great resource for genetic elements hosting stress-tolerant genes. A mechanism of HGT allows the sharing of such elements among diverse microbial populations. The HGT is unlikely to be a one-step process but requires genome innovation and the building of a regulatory network for the functionalization of transferred genes [11]. Further research into this area would help equip vulnerable hosts to diversify under less-than-ideal environmental conditions. Legume plantations enrich the soil with organic nitrogen, which reduces the synthetic N-fertilizers input in subsequent crops and helps prevent nitrogen pollution. Although soil microbiomes evolve to adjust to changing conditions, the loss of diversity occurs when pollutants, either in the form of fertilizers, pesticides, or industrial waste, enter the soil. Preserving the microbiome’s diversity is important to maintain the health of the ecosystem, which has been correlated with plant productivity [166].
